# Loss of *Nfkb1* leads to early onset aging

**DOI:** 10.18632/aging.100702

**Published:** 2014-12-01

**Authors:** Giovanna M. Bernal, Joshua S. Wahlstrom, Clayton D. Crawley, Kirk E. Cahill, Peter Pytel, Hua Liang, Shijun Kang, Ralph R. Weichselbaum, Bakhtiar Yamini

**Affiliations:** ^1^ Department of Surgery, Section of Neurosurgery, University of Chicago, Chicago, IL 60637, USA; ^2^ Department of Pathology, University of Chicago, Chicago, IL 60637, USA; ^3^ Department of Radiation and Cellular Oncology and the Ludwig Center for Metastasis Research, University of Chicago, Chicago, IL 60637, USA

**Keywords:** Nfkb1, Aging, CNS gliosis, γH2AX, Replicative senescence

## Abstract

NF-(B is a major regulator of age-dependent gene expression and the p50/NF-(B1 subunit is an integral modulator of NF-(B signaling. Here, we examined *Nfkb1^−/−^* mice to investigate the relationship between this subunit and aging. Although *Nfkb1^−/−^* mice appear similar to littermates at six months of age, by 12 months they have a higher incidence of several observable age-related phenotypes. In addition, aged *Nfkb1^−/−^* animals have increased kyphosis, decreased cortical bone, increased brain GFAP staining and a decrease in overall lifespan compared to *Nfkb1^+/+^. In vitro*, serially passaged primary *Nfkb1^−/−^* MEFs have more senescent cells than comparable *Nfkb1^+/+^* MEFs. Also, *Nfkb1^−/−^* MEFs have greater amounts of phospho-H2AX foci and lower levels of spontaneous apoptosis than *Nfkb1^+/+^*, findings that are mirrored in the brains of *Nfkb1^−/−^* animals compared to *Nfkb1^+/+^*. Finally, in wildtype animals a substantial decrease in p50 DNA binding is seen in aged tissue compared to young. Together, these data show that loss of *Nfkb1* leads to early animal aging that is associated with reduced apoptosis and increased cellular senescence. Moreover, loss of p50 DNA binding is a prominent feature of aged mice relative to young. These findings support the strong link between the NF-(B pathway and mammalian aging.

## INTRODUCTION

Aging is a universal process involving the progressive decline in organ function that eventually leads to organismal death. While accumulation of DNA damage has long been considered the central cause of aging [1], more recent observations suggest that aging is the result of a continuation of early-life hyperfunction programs [2-4]. Regardless of mechanism, cellular senescence is a central finding associated with mammalian aging [5], an observation emphasized by a report demonstrating that apoptotic removal of senescent cells preserves tissue homeostasis and extends overall animal health [6, 7]. Nuclear factor-(B (NF-(B) is a ubiquitously expressed transcription factor that has been intimately linked to cellular senescence, DNA damage signaling and organismal aging [8-11].

The NF-(B family of proteins consist of five subunits: p50 (NF-(B1, p105), p52 (NF-(B2, p100), p65 (relA), c-rel, and relB, that appear in their mature form as dimers [12]. In unstimulated cells, NF-(B dimers are retained in the cytoplasm through interaction with inhibitor-κB (IκB) proteins. NF-(B activation occurs through a number of related pathways that, in general, converge on the cytoplasmic IκB kinase (IκK) complex. Activation of IκK leads to IκB protein degradation and subsequent NF-(B nuclear translocation. In the nucleus, NF-(B dimers regulate genes involved in a wide range of cellular processes. While the most abundant form of NF-(B is comprised of the p50/p65 heterodimer, p50 homodimers predominate in many unstimulated cells and tissues. The ubiquitous expression of p50 suggests that this subunit plays a critical role in a broad range of physiological processes. p50 is encoded by the *NFKB1* gene and is produced from the N-terminus of NF-(B1/p105 following proteosomal processing. Mice deleted of *Nfkb1* are viable and despite having specific defects in innate and adaptive immunity [13], were originally noted to have a normal lifespan up to 1 year [14].

We recently demonstrated that p50 (NF-(B1/p105) is an effector protein that mediates the apoptotic response to S-phase DNA damage and replication stress [15]. This observation suggests that p50/NF-(B1 may act physiologically to maintain overall animal health. To examine this hypothesis, we followed cohorts of *Nfkb1^−/−^* mice and their littermate controls and find a distinct propensity for early onset of age-related pathology with loss of *Nfkb1*. In addition, in both tissue specimens and cultured primary cells, loss of *Nfkb1* leads to an increase in cellular senescence and a decrease in spontaneous apoptosis. These data indicate that *Nfkb1* acts to maintain animal longevity and, together with the observation that p50 DNA binding is lost in aged compared to young tissue, suggest that loss of this NF-(B subunit is associated with physiological aging.

## RESULTS

### Loss of *Nfkb1* accelerates observable age-related characteristics and leads to a decrease in lifespan

The importance of p50/NF-(B1 in mediating apoptotic signaling raised the question of whether loss of this subunit leads to a predisposition for the development of chronic disease. We therefore followed cohorts of *Nfkb1^+/+^* and *Nfkb1^−/−^* animals over an extended period of time. While the most commonly used *Nfkb1^−/−^* mouse is found on the original B6/129 cross background [14], it is well documented that animal strain plays a significant role in lifespan and disease predisposition [16]. Therefore, we obtained *Nfkb1^−/−^* animals that have been backcrossed to C57BL6 mice for 12 generations and interbred them with wildtype (wt) C57BL6 mice to obtain single strain littermates. Because of the susceptibility of *Nfkb1^−/−^* mice to infection when housed under normal conditions [14], animals were followed in a pathogen-free environment and sacrificed when they displayed signs associated with a terminal state.

**Figure 1:**
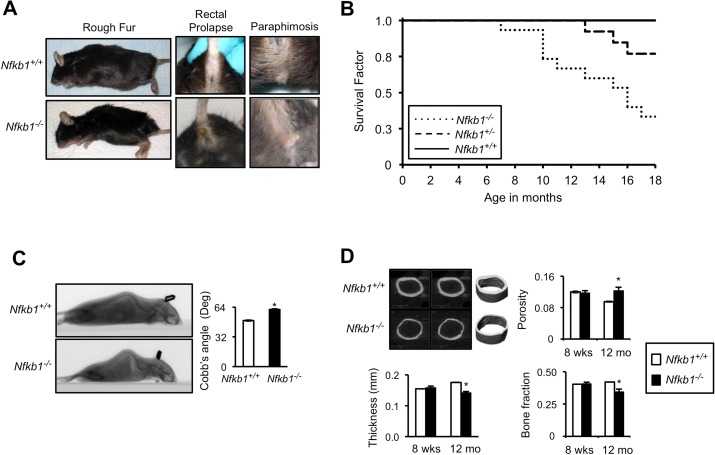
*Nfkb1^−/−^* mice have accelerated age-related findings and reduced lifespan (**A**) Representative images of superficial age-related characteristics in *Nfkb1^−/−^* and *Nfkb1^+/+^* mice at 18 months of age. (**B**) Kaplan-Meier survival curves of *Nfkb1^+/+^* (n=11), *Nfkb1^+/−^* (n=13) and *Nfkb1^−/−^* (n=15) littermate mice. p<0.01, *Nfkb1^+/+^ vs. Nfkb1^−/−^* and p<0.05, *Nfkb1^+/−^ vs. Nfkb1^−/−^*, Log rank. (**C**) Lateral spiral CT images (left) of 12-month old *Nfkb1^−/−^* and *Nfkb1^+/+^* mice. Quantification of Cobb's Angle (right), in degrees, based on lateral CT image calculated as described in the methods (n=6 for *Nfkb1^−/−^* and n=11 for *Nfkb1^+/+^*). (**D**) Cortical μCT imaging of femurs from *Nfkb1^−/−^* and *Nfkb1^+/+^* animals. Representative images of 12-month old femurs are shown. Graphs represent mean cortical thickness (mm), cortical porosity, and cortical bone fraction of 8-week and 12-month old animals (n=3 per group in 8-week old animals and n=4 per group in 12-month old). Data represent mean +/− SEM. *p<0.05.

Consistent with the original description of these animals, other than being slightly smaller than wt, *Nfkb1^−/−^* mice are identical to their littermates and do not display any overt differences for the first six months of life. However, at 12 and 18 months, compared to age-matched *Nfkb1^+/+^* animals, *Nfkb1^−/−^* mice have a higher incidence of several observable age-related characteris-tics including rough fur coat, alopecia, rectal prolapse and paraphimosis (Fig. 1A, Table 1).

**Table 1:** Age-related phenotypes in *Nfkb1^+/+^* and *Nfkb1^−/−^* mice

	12-month old		18-month old	
	*Nfkb1^+/+^*	*Nfkb1 ^−/−^*	*Nfkb1^+/+^*	*Nfkb1 ^−/−^*
	Female	Female	Male	Male
Rough Fur Coat	0/14 (0%)	3/8 (38%)*	3/6 (50%)	7/7 (100%)*
Alopecia	4/14 (29%)	2/8 (25%)	1/6 (17%)	4/7 (57%)
Rectal Prolapse	0/14 (0%)	1/8 (13%)	0/6 (0%)	3/7 (43%)#
Paraphimosis	-	-	2/6 (33%)	3/7 (43%)

Animal numbers: 12-month old: *Nfkb1*^+/+^ n=14, *Nfkb1*^−/−^ n=9; 18-month old: *Nfkb1*^+/+^ n=6, *Nfkb1*^−/−^ n=7.

* p<0.05, Chi-squared.

# p=0.06.

These observations suggest that loss of *Nfkb1* leads to premature aging. Consistent with this hypothesis, when *Nfkb1^+/+^*, *Nfkb1^+/−^* and *Nfkb1^−/−^* littermate mice are followed for eighteen months, significantly fewer *Nfkb1^−/−^* mice remain alive than *Nfkb1^+/+^* (Fig. 1B). While all *Nfkb1^+/+^* animals are alive at 1 year, only 70 % of *Nfkb1^−/−^* mice remain alive at this time (*P<*0.01, Log rank). Moreover, heterozygote mice have an intermediate survival that is significantly greater than that of *Nfkb1^−/−^* mice (p<0.05, Log rank). Notably, autopsy reveals no evidence of overwhelming infection or sepsis in the *Nfkb1^−/−^* animals nor do they have evidence of increased tumor formation compared to controls.

### Loss of *Nfkb1* leads to premature age-related skeletal changes

Given the findings with loss of *Nfkb1,* we next examined whether *Nfkb1^−/−^* animals also have premature age-related skeletal changes. Gross inspection suggests that compared to *Nfkb1^+/+^* mice, *Nfkb1^−/−^* animals have an in-crease in kyphosis (Fig. 1A), a finding closely associated with advanced age [17]. To more objectively examine kyphosis, spiral CT was performed on 12-month old *Nfkb1^−/−^* and *Nfkb1^+/+^* animals and Cobb's angle measured (Fig. 1C). *Nfkb1^−/−^* mice have significantly higher Cobb's angle (p<0.05), confirming that loss of *Nfkb1* is associated with increased kyphosis. Age-related kyphosis has been linked to osteoporosis and decreased cortical bone density [18]. We therefore examined cortical bone density using high-resolution micro–computed tomography (μCT) imaging of femurs. Although at 8 weeks there is no difference in cortical bone characteristics between *Nfkb1^+/+^* and *Nfkb1^−/−^* animals, by 12 months *Nfkb1^−/−^* mice have a significantly lower cortical thickness and cortical bone fraction (p<0.05) and higher cortical porosity (p=0.05) than *Nfkb1^+/+^* (Fig. 1D). These findings further support the hypothesis that loss of *Nfkb1* leads to premature mouse aging.

### Loss of *Nfkb1* results in tissue inflammation and increased CNS gliosis

**Figure 2:**
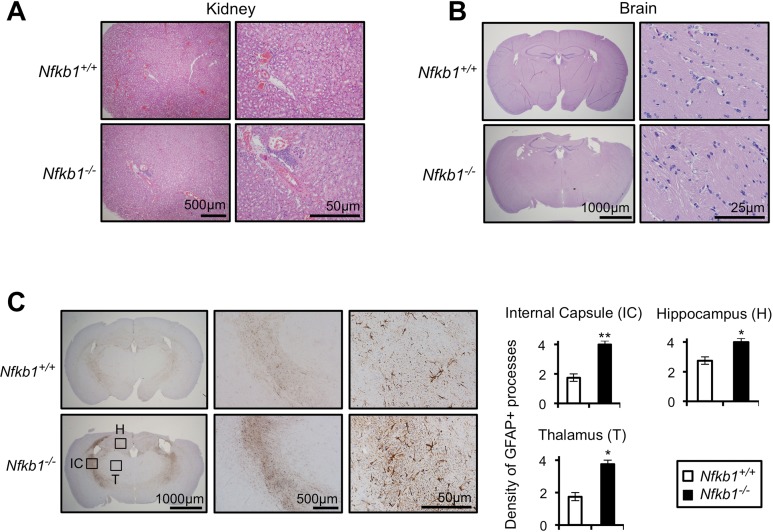
*Nfkb1^−/−^* mice exhibit enhanced age-related CNS gliosis Representative H&E stained kidney (**A**) and brain (**B**) sections from 12-month old *Nfkb1^+/+^* and *Nfkb1^−/−^* mice (n=4 per group). (**C**) Representative brain sections (left) from 12-month old *Nfkb1^+/+^* and *Nfkb1^−/−^* mice stained with anti-GFAP antibody. IC-internal capsule, H-hippocampus, T-thalamus. Graphs (right) demonstrate density of GFAP processes within the indicated region determined by averaging the level of staining from three separate fields in four coronal sections, separated by 3 mm (A-P diameter). n=4 animals per group. Scale: 1- very few GFAP processes; 2: <25% staining; 3: 25-50% staining and 4: >50% staining. Data represent mean +/− SEM. *p<0.03. **p<0.01.

We next performed histological analysis of tissues, and consistent with a previous report that *Nfkb1^−/−^* mice have increased inflammatory cell infiltrate in the liver [19], we note an increase in inflammation in the kidney (Fig. 2A) and skeletal muscles (data not shown) of *Nfkb1^−/−^* compared to *Nfkb1^+/+^* mice. Examination of age-matched brain sections does not reveal differences in inflammation or other histomorphologic features (Fig. 2B). However, given the association between aging and CNS white matter glial fibrillary acidic protein (GFAP) staining [20], we examined GFAP reactivity in these animals. A significantly higher level of GFAP labeling is observed in brains of 18 month-old *Nfkb1^−/−^* animals compared to *Nfkb1^+/+^* (p<0.01, Fig. 2C). These findings confirm the increase in inflammation in *Nfkb1^−/−^* animals and also demonstrate that these mice have increased CNS gliosis relative to age-matched *Nfkb1^+/+^* littermates.

Taken together, the above results indicate that loss of *Nfkb1* leads to the premature appearance of multiple age-related findings underlined by a decrease in overall lifespan.

### Loss of *Nfkb1* results in premature cellular senescence

**Figure 3:**
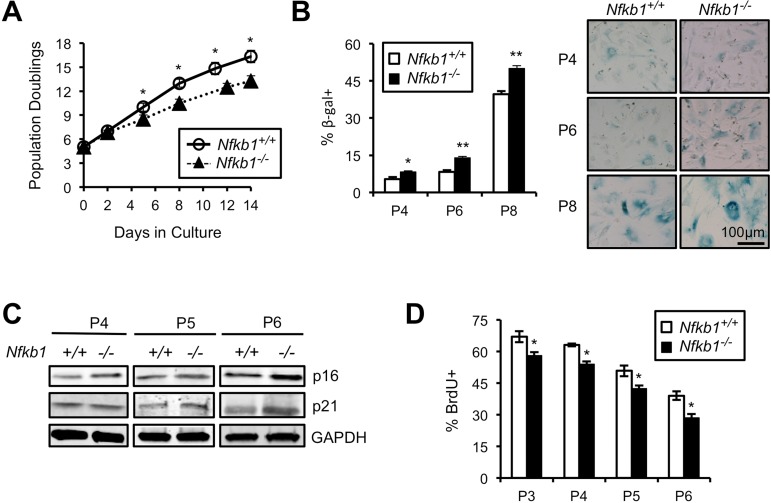
Primary *Nfkb1^−/−^* MEFs exhibit premature cellular senescence (**A**) Growth curves of primary *Nfkb1^−/−^* and *Nfkb1^+/+^* MEFs (n=3 separate MEF isolates per group, harvested at different times) starting at PD5 after harvest (set as time 0). (**B**) SA-β-gal staining in *Nfkb1^−/−^* and *Nfkb1^+/+^* MEFs at the indicated passage. n=3 per group. Representative image shown (right). (**C**) Immunoblot using anti-p16 and anti-p21 antibody in primary *Nfkb1^−/−^* and *Nfkb1^+/+^* MEFs at passage 4, 5 and 6. (**D**) Quantification of percent BrdU-positive cells at the indicated passage. n=3 isolates per group, harvested at different times. Data represent mean +/− SEM. *p<0.05; **p<0.01.

Primary cells have a limited lifespan in culture and undergo senescence following serial passage [5]. This observation, coupled with the accelerat ed aging of *Nfkb1^−/−^* mice raises the question of whether loss of *Nfkb1* affects the lifespan of cells *in vitro*. We isolated primary mouse embryonic fibroblasts (MEFs) from *Nfkb1^+/+^* and *Nfkb1^−/−^* animals and examined their growth in culture. A significant decrease in population doubling (PD) rate is seen in *Nfkb1^−/−^* MEFs compared to *Nfkb1^+/+^* with increasing time in culture (Fig. 3A). Importantly, this observation is noted using multiple separate MEF isolates harvested at different times. The decrease in *Nfkb1^−/−^* cell number is associated with an increase in cellular senescence, relative to *Nfkb1^+/+^*, as demonstrated by a change in cellular morphology to an enlarged and flattened shape and an increase in senescence associated-β-galactosidase (SA-β-gal) staining (Fig. 3B). In addition, we studied expression of the cyclin-dependent kinase inhibitors, p21 and p16 (CDKN1a and CDKN2a, respectively), factors that are closely associated with senescence [21]. Although both cell types have relatively equal expression of p21 and p16 initially, at later passage the expression of both proteins is higher in *Nfkb1*^−/−^ MEFs than similarly passaged *Nfkb1^+/+^* cells (Fig. 3C). Finally, to compare the rate of proliferation, S-phase-dependent incorpora-tion of the thymidine analog 5-bromodeoxyuridine (BrdU) was examined. *Nfkb1^−/−^* MEFs have less BrdU-positive cells than *Nfkb1^+/+^* cells at a similar passage (Fig. 3D). Together, these results suggest that loss of *Nfkb1* leads to a decline in PD rate due to an increase in senescence.

### Loss of *Nfkb1* leads to reduced apoptosis and increased phospho-H2AX accumulation

**Figure 4:**
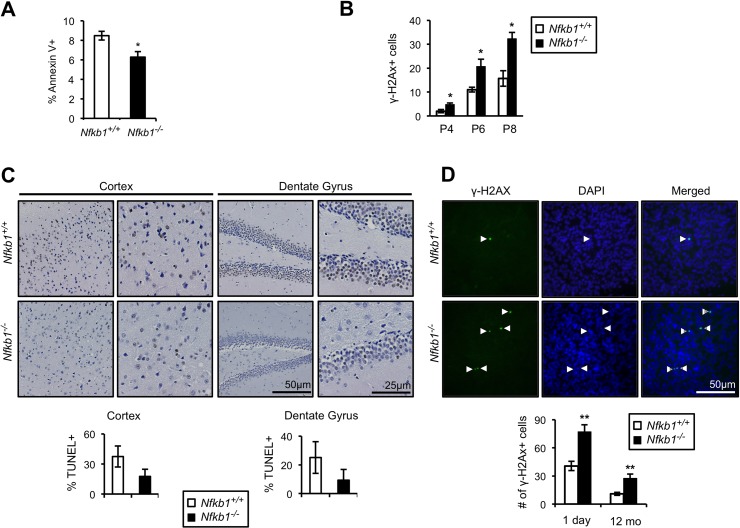
Loss of *Nfkb1* is associated with decreased apoptosis and increased γH2AX accumulation (**A**) Annexin V binding in primary *Nfkb1^+/+^* and *Nfkb1^−/−^* MEFs at passage 2 (n=5 MEF isolates per group, harvested at different times). (**B**) γH2AX staining in primary *Nfkb1^+/+^* and *Nfkb1^−/−^* MEFs at the indicated passage. MEF data show mean +/− SD. (**C**) Percentage of cells that are TUNEL-positive in the cortex and dentate gyrus in 12-month old *Nfkb1^+/+^* and *Nfkb1^−/−^* mice (n=4 animals per group). Representative sections shown (above). (**D**) Total number of γH2AX foci in brain sections from newborn and 12-month old *Nfkb1^+/+^* and *Nfkb1^−/−^* mice (n=3 animals per group). γH2AX positive cells were calculated as described in the methods, data represent mean +/− SEM. Representative coronal brain sections demonstrate co-localization of γH2AX and DAPI (arrow heads). *p<0.05. **p<0.01.

An association between cellular senescence and loss of apoptosis is well described [22]. Given that loss of *Nfkb1* leads to decreased apoptosis in response to DNA alkylation damage [15] and that alkylation damage is induced by environmental and endogenous processes [23, 24], we examined spontaneous apoptosis in cultured MEFs. Primary *Nfkb1^+/+^* and *Nfkb1^−/−^* MEFs were harvested and apoptosis levels assessed by analysis of annexin V binding. *Nfkb1^−/−^* MEFs have a significantly lower level of spontaneous apoptosis than *Nfkb1^+/+^* MEFs at the same passage (p<0.05; Fig. 4A), an observation noted with multiple separate MEF isolates. Cellular senescence and animal aging have been linked to expression of markers of activation of the DNA damage response including phosphorylation of histone H2AX (γH2AX) [25-27]. Therefore, we examined MEFs for γH2AX foci formation. *Nfkb1^−/−^* MEFs accumulate significantly more γH2AX foci than *Nfkb1^+/+^* following serial passage (p<0.05, Fig. 4B, Fig. Supplemental S1), indicating that loss of *Nfkb1* leads to an increase in damage signaling.

The difference in spontaneous apoptosis and γH2AX foci in cultured cells raises the question of whether such changes are also evident *in vivo*. To examine apoptosis *in vivo*, we looked at TUNEL and caspase 3 staining in brain sections of 12-month old *Nfkb1^+/+^* and *Nfkb1^−/−^* mice. Consistent with the MEF data, *Nfkb1^+/+^* mice have substantially more apoptotic cells in the cortex and dentate gyrus than *Nfkb1^−/−^* mice as demonstrated by both TUNEL analysis (Fig. 4C) and caspase 3 staining (Fig. Supplemental S2). In addition, examination of γH2AX foci in both newborn and 12-month old mouse brains demonstrates that, as with MEFs, significantly more γH2AX foci are seen in the brains of *Nfkb1^−/−^* compared to age-matched *Nfkb1^+/+^* animals (p<0.01, Fig. 4D).

These findings indicate that loss of *Nfkb1* leads to a decrease in spontaneous apoptosis and an increase in DNA damage signaling, findings that are consistent with the increase in cellular senescence and premature aging seen in *Nfkb1^−/−^* cells and animals.

### Aging is associated with a relative decrease in p50 DNA binding

**Figure 5:**
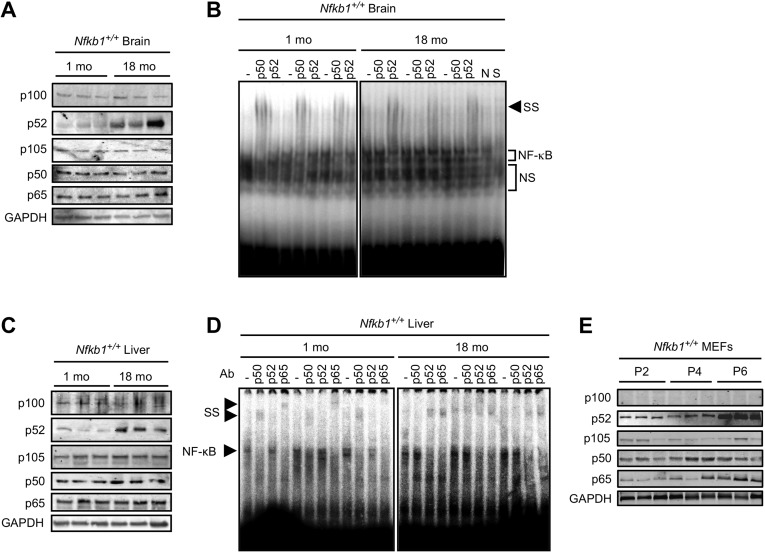
p50 DNA binding declines with age (**A** and **C**) Immunoblots, using the indicated antibody, in tissue lysate from *Nfkb1^+/+^* mouse brain (**A**) and liver (**C**) at 1 and 18 months of age. n=3 animals per group. (**B** and **D**) EMSA in tissue lysate from *Nfkb1^+/+^* mouse brain (**B**) and liver (**D**) at 1 and 18 months of age. Supershift (SS) was performed with the indicated antibodies. n=3 animals per group. N-non-specific and S-specific cold competitors verify the NF-(B bands. NS-non-specific bands. (**E**) Immunoblot using the indicated antibodies in primary *Nfkb1*^+/+^ MEFs at passage 2, 4, and 6. n=3 each lane represents a separate MEF isolate.

The premature aging of *Nfkb1^−/−^* mice raises the question of whether loss of *Nfkb1* is associated with physiological aging. To study this question, we looked at NF-(B subunit expression in young and old *Nfkb1^+/+^* brains. Although there is no change in p50 or p105 protein levels, a substantial increase in p52, the other non-rel NF-(B subunit, is noted in 18-month old compared to 1-month old brain tissue (Fig. 5A). The age-dependent increase in p52 is a progressive process as 6-month old animals have an intermediate level of p52 expression (Fig. Supplemental S3A). Minimal increase in p100 and p65 level is also noted in the aged brains (Fig. 5A). Interestingly, as with *Nfkb1^+/+^* tissue, an increase in p52 is also seen in aged compared to young *Nfkb1^−/−^* brains (Fig. Supplemental S3B).

NF-(B mediates its action by binding DNA, therefore to examine whether the increase in p52 is associated with a change in the composition of the DNA bound NF-(B dimer, gel shift assay was used. As previously reported with other tissue [28-30], a general increase in NF-(B DNA binding is evident in brains of old mice compared to young (Fig. Supplemental S3C). Remarkably, however, supershift analysis consistently demonstrates that while anti-p50 shifts the NF-(B band in young brains (Fig. 5B), in old brain anti-p50 has no effect and instead anti-p52 induces supershift. These results indicate that p52 replaces p50 in the DNA bound dimer in aged brain compared to young. We next examined whether the change in NF-(B dimer composition is also present in other tissue. Consistent with the findings in the brain, an increase in p52, but not p50, protein is seen in the liver of aged compared to young *Nfkb1^+/+^* mice (Fig. 5C). Similarly, aged *Nfkb1^−/−^* mice have higher p52 protein levels in their liver than young animals (Fig. Supplemental S3D). Moreover, supershift analysis recapitulates the findings in brain tissue showing that while p50 binds DNA in young liver, it is replaced by p52 in the aged tissue (Fig. 5D). Supershift analysis also demonstrates that p65 remains part of the NF-(B dimer in both young and old tissue, consistent with previous observations in cells demonstrating that in the absence of p50, p52 dimerizes with p65 [15, 31].

Finally, we examined whether cultured cells also demonstrate changes in subunit levels with increasing passage. Although no change in p50 or p105 expression is noted in *Nfkb1^+/+^* MEFs over serial passage, a robust increase in p52, but not p100, is seen at late passage (Fig. 5E). Similarly, in *Nfkb1^−/−^* MEFs an increase in p52, but not p100, is seen in late compared to early passage (Fig. Supplemental S3E).

Taken together, the findings in old compared to young tissue indicate that p50 is replaced by p52 in aged tissue and that the overall increase in NF-(B binding with age is related to an increase in p52. These findings suggest that loss of DNA bound p50 is a prominent feature of physiological aging.

## DISCUSSION

Increasing evidence points to the NF-(B pathway as being a primary regulator of mammalian aging [32, 33]. However, although p50/NF-(B1 makes-up a significant portion of the basal NF-(B dimer, the role of this subunit in age-related pathology has been under examined. By following a colony of *Nfkb1^−/−^* mice over an extended period of time, we demonstrate that loss of *Nfkb1* leads to the early appearance of multiple age-related findings accentuated by a decrease in overall lifespan. Importantly, the early aging of *Nfkb1^−/−^* mice appears to be a physiologic process, and not just a shortening of lifespan, as these mice are normal when young and develop age-related findings at an appropriate time in their overall survival curve. While these findings indicate that loss of NF-(B accelerates aging, previous studies note the reverse, that inhibition of NF-(B actually delays certain age-related findings [8, 29]. These contradictory observations are not mutually exclusive and are likely related to the subunit-specific effects of the different NF-(B proteins [13]. Whereas studies that show that NF-(B promotes aging primarily implicate either p65 or NF-(B in general, the current work is related specifically to *Nfkb1*. Moreover, loss of *Nfkb1* is not commensurate with inhibition of NF-(B as *Nfkb1^−/−^* cells retain significant levels of DNA bound NF-(B and also have a similar NF-(B-dependent response to stimulation as wildtype cells [15, 31].

The original description of *Nfkb1^−/−^* mice did not comment on early aging for animals housed in a pathogen-free environment [14]. However, more recent studies have noted several age-related findings in these animals. Premature death was suggested, though not further substantiated, by one group who noted that 25 % of their *Nfkb1^−/−^* mice died by 4 months of age [34]. This study also reported an increase in age-related neural degeneration with loss of *Nfkb1*. Age-related CNS findings in *Nfkb1^−/−^* mice have also been noted by others including hearing loss and spiral ganglion cell depletion [35], retinal ganglion cell loss [36] and short-term spatial memory deficits [37]. In this latter study, it was specifically noted that there is a decrease in late maturation of *Nfkb1^−/−^* hippocampal neurons. The increase in age-related CNS findings in *Nfkb1^−/−^* animals is consistent with our observation of increased GFAP reactivity and γH2AX foci in the brains of *Nfkb1^−/−^* mice. While the mechanistic link between GFAP staining and aging remains unclear, a strong correlation between these findings is evident both in rodents and humans [20, 38, 39]. Also, when studying age-related neurodegenerative changes, the importance of animal strain cannot be underestimated. Although we have endeavored to minimize this by using single strain littermate mice, C57BL/6 mice have been reported to have early onset of certain neurodegenerative findings including hearing loss [40] that may contribute to some of the observations noted in our study. Nevertheless, other organs have also been implicated in the premature aging of *Nfkb1^−/−^* mice as exemplified both by our results and the early emphysema and decreased skin thickness noted by others [41, 42].

At the cellular level, loss of *Nfkb1* results in reduced apoptosis, increased senescence and increased γH2AX focus formation. While a direct link between cellular senescence and organismal aging has not been definitively established, aged tissues have higher levels of senescent cells than young [43]. Moreover, a recent study demonstrated that apoptotic removal of senescent cells delays the appearance of certain age-related findings [6]. We have found that p50/NF-(B1 mediates the apoptotic response to replication stress and that loss of this pathway results in cellular senescence and genome instability (ref [15] and unpublished data, BY). These findings, when considered with the well-described association between senescence, resistance to apoptosis and organismal aging [22, 44], suggest that *Nfkb1* maintains organism health, at least in part, via its role in the response to endogenous replication stress. In studying *Nfkb1^−/−^* mice, it is important to appreciate that these animals have specific deficits in innate and adaptive immunity [13, 14]. However, when housed in a pathogen-free environment, these animals do not have overt signs of systemic infection and they do not die early from an infectious etiology. Interestingly, aging is associated with a general increase in tissue inflammation [45], a finding that has been linked to a hyper-function program [2-4], and the increase in NF-(B binding in aged tissue is thought to contribute to this inflammatory phenotype [32, 33]. *Nfkb1^−/−^* mice have increased inflammation in several tissues as noted by others [19] and us. While the immune deficiency of *Nfkb1^−/−^* mice likely contributes to early aging, the observation that loss of *Nfkb1* leads to decreased apoptosis, increased γH2AX focus formation and increased senescence not only in tissues but also in cultured cells, suggests that age-related findings with loss of this subunit are at least in part cell autonomous. An important question raised by the premature aging of *Nfkb1^−/−^* mice concerns the role of this subunit in physiological aging. To study this issue, we examined the make-up of the NF-(B dimer in young and old animals. NF-(B dimer composition changes with age such that while p50 binding is present in young tissue, in old animals p50 binding is lost and replaced by p52. Although age-associated increase in overall NF-(B binding has been described [28-30, 46-49], a change in dimer composition has not previously been reported. While p50 protein level remains stable with advancing age, p52 increases, a finding also noted in rat liver and gastric mucosa [47, 50]. These observations suggest that the change in NF-(B dimer composition occurs as a result of increased p52. However, despite the functional redundancy of NF-(B subunits [31], p52 cannot compensate for p50 in the response to S-phase damage [15]. In sum, as depletion of p50 (by gene knockout or si-RNA) results in reduced cellular apoptosis and increased senescence, it is tempting to hypothesize that the natural decrease in p50 DNA binding that occurs physiologically with age contributes to the increase in cellular senescence that ultimately promotes aging.

The data presented demonstrate that deletion of *Nfkb1* results in reduced apoptosis increased cellular senescence and accelerated aging. Also, examination of tissues from *Nfkb1^−/−^* mice reveals an increase in inflammation, osteoporosis, damage signaling and gliosis compared to wildtype, findings that are consistent with a previously described hyperfunction program [2, 4]. Together, these observations add further complexity to the role of NF-(B in the aging process and indicate that caution should be taken when considering general NF-(B manipulation for the treatment of chronic disease. In addition, the results suggest that elucidating the mechanism by which p50 and p52 DNA binding changes over time may potentially uncover novel pathways that can be targeted to ameliorate specific age-related diseases.

## METHODS

### Animals

*Nfkb1^−/−^* animals that have been backcrossed for 12 generations with C57BL/6J mice (B6.Cg-*Nfkb1^tm1Bal^*/J), and the corresponding C57BL/6J *Nfkb1^+/+^* control, were obtained (Jackson Laboratory) and kept in a pathogen-free environment with food and water available ad libitum and a 12-hr light/dark cycle. Mice were interbred and the resultant *Nfkb1^+/+^*, *Nfkb1^+/−^* and *Nfkb1^−/−^* littermates used for long-term studies. All animals were maintained in accordance to a protocol approved by the Institutional Animal Care and Use Committee of the University of Chicago.

### Generation and culture of MEFs

MEFs were harvested from 13-14 day *Nfkb1^+/+^* and *Nfkb1^−/−^* embryos. Briefly, head and visceral organs were removed and remaining tissue chopped in cold, sterile PBS. The tissue was digested in 0.25% trypsin for 5 minutes at 37°C and then dispersed and spun down with fetal bovine serum (FBS). The supernatant was collected and spun down with high glucose DMEM supplemented with 10% FBS and 5% of a penicillin and streptomycin (Pen/Strep) cocktail. The cell pellet was then plated with DMEM and 20% FBS on a 10 cm dish. MEFs were slowly weaned off FBS with daily media changes over the next few days to reach a final concentration of 10% FBS. Subsequently, cells were passaged with each passage considered to be approximately 2 PDs. To examine growth curves, cells were plated starting between passage 2 and 3 (estimated to be PD5) at 25% confluency and passaged when they achieved approximately 75% confluency. Cells were passaged until they reached crisis and looked unhealthy.

### SA-β-gal and γH2AX analysis

Primary MEFs at the indicated passage were plated onto 35 mm glass bottom plates (MatTek) and at 60 % confluency were fixed with formaldehyde for 20 minutes at 4°C. SA-β-gal staining was performed in the standard fashion. Cells were incubated overnight with freshly made staining solution and the percentage of cells positive for SA-β-gal was calculated from a total of 200 cells per plate. For γH2AX analysis, cells were incubated in rabbit anti-γH2AX antibody (Cell Signaling) for one hour followed by goat anti-rabbit FITC secondary antibody (Vector Laboratories Inc., Burlingame, CA, USA). Glass bottom coverslips were removed and mounted on slides using Fluoro Gel-II mounting medium containing DAPI (Electron Microscopy Sciences). Experiments for SA-β-gal and γH2AX analysis were repeated in triplicate.

### Flow cytometry for annexin V binding and BrdU analysis

Annexin V and BrdU were analyzed using flow cytometry. Briefly, for detection of annexin V, cells were incubated with FITC-conjugated anti-annexin V, followed by propidium iodide (BD Biosciences), and immediately analyzed. For detection of BrdU, MEFs were incubated with 0.03 mg/ml BrdU for 1 hour, fixed in ice-cold methanol for 5 minutes and treated with 1.5 M HCl for 30 minutes at room temperature. Following blocking, cells were incubated with anti-BrdU monoclonal antibody (Santa Cruz) in 0.1% BSA for 1 hour. Cells were then incubated in donkey anti-mouse APC-conjugated secondary antibody (Millipore) for 30 minutes. Flow cytometry was performed using the BD LSR II (BD Biosciences) equipped with a 635-nm laser. Experiments were performed using at least 3 individually harvested cell populations per group, and each sample was run in triplicate. Data was analyzed using FlowJo software (TreeStar Inc.).

### Cobb's angle measurement

To measure kyphosis, lateral images from a spiral CT scan of the spine of 12-month-old mice were acquired. Cobb's angle was calculated in the standard fashion by measuring the angle formed by the juncture of two parallel lines drawn between the last two vertebra found at the most rostral and caudal ends of the curvature. Images were analyzed using ImageJ (National Institutes of Health).

### μCT analysis of femurs

Femurs were harvested from 8-week and 12-month old mice, cleaned of soft tissue and placed in a 20.5 mm tube. Samples were scanned horizontally, approximately 300 slices per sample, with a desktop cone-beam μCT scanner (SCANCO Model μCT40; SCANCO Medical). Femurs were then rotated to transverse slices and analyzed. Cortical bone was measured at the midshaft (50 % of the length of the femur), and then 50 slices were included on either side (100 slices total). Contours for cortical bone were drawn using the midshaft contour script (#16), and each was inspected & modified for best fit before analysis. Cortical bone was analyzed using μCT Evaluation Program V6.0 (SCANCO Medical) with the midshaft cortical bone script (#23) with multiple thresholds to gauge best fit, and a threshold of 350 was selected to be the most appropriate.

### Histology, TUNEL staining and immunohistochemistry

For general brain histology, GFAP and caspase-3 immunohistochemistry, mice were sacrificed and brains and other tissue harvested and fixed in formalin. The tissue was processed for paraffin embedding and sectioned at 4 μm. Tissue slides were deparaffinized for hematoxylin and eosin staining, or incubated in either rabbit anti-GFAP antibody (DAKO) or rabbit anti-caspase-3 antibody (Millipore) overnight. GFAP and caspase-3 slides were then incubated with goat anti-rabbit secondary antibody (DAKO), and counterstained with hematoxylin. For γH2AX immunohistochemistry, fixed brain tissue from newborn or 12-month old mice was frozen and sectioned at 20 μm with a cryostat. Sections were incubated in rabbit anti-γH2AX (Cell Signaling) overnight followed by incubation with a goat anti-rabbit FITC secondary antibody (Vector Laboratories). Slides were coverslipped with Fluoro Gel-II mounting medium containing DAPI and analyzed using the Axiovert 200M inverted widefield fluorescence microscope (Zeiss). Counting for γH2AX-positive cells in brain tissue was performed in blinded fashion on a total of 9 sections per animal separated by a minimum distance of 150 μm. All γH2AX-positive cells were counted in each section under analysis and the total number of cells from all 9 sections reported. For TUNEL analysis, the Apoptosis Detection Kit was used according to the manufacturer's instructions (Millipore). Sections were counterstained with hematoxylin and the ratio of TUNEL-positive cells to TUNEL-negative cells was calculated from 3 random areas within each specified brain region (internal capsule, hippocampus and thalamus) under bright microscopy at a magnitude of 40x. Data represent the average number of positive cells counted in sections from three separate animals in each group.

### Immunoblotting and electrophoretic mobility shift assay (EMSA)

For immunoblot analysis, harvested tissue was flash frozen and manually triturated upon thawing. Both tissue and MEFs were then lysed with RIPA buffer and the resulting protein boiled with Laemmli buffer and loaded on a 4-15% polyacrylamide gel. Following electrophoresis (1.5 h at 150V), protein was electrotransferred onto nitrocellulose membranes overnight. The following day, membranes were blocked in 1% milk with TBS and 0.1% Tween 20 for 2 hrs and then incubated overnight with the indicated antibodies (GAPDH from Cell Signaling; all other antibodies are from Santa Cruz). Membranes were then rinsed with buffer and incubated with fluorescent-tagged secondary antibodies (Thermo Scientific) for 2 hours. After rinsing, membranes were analyzed by computer-assisted image analysis (LI-COR Biosciences), with comparisons made between samples on the same gel/immunoblot.

For EMSA, frozen mouse tissue was rinsed in PBS buffer and spun at 1,500 x g for 5 min. Tissue was homogenized in extraction buffer (20 mM HEPES, pH 7.5, 100mM NaCl, 0.05% Triton X-100, 1 mM dithiothreitol (DTT), 5 mM sodium b-glycophosphate, 0.5 mM sodium orthovanadate, 1 mM EDTA, 0.5 mM phenylmethylsulfonyl fluoride (PMSF), 10 μg/mL aprotinin, 5 μg/mL leupeptin, 2 μg/mL pepstatin) with pestle on ice. Lysate was cleared by centrifugation at 12000g for 15 min at 4**°**C. EMSA was performed using 5 μg of extract and a ^32^P-labeled double-stranded 30-mer oligonucleotides containing a single central κB-site (GGGACTTTCC) with ten flanking base pairs on either side as a probe. EMSA reactions contained 10 mM Tris-HCl (pH 7.5), 1 mM MgCl_2_, 50 mM NaCl, 0.5 mM EDTA, 0.5 mM DTT, 4% glycerol, 50 ng/μl poly(dI·dC). Reaction mixtures were incubated at room temperature for 20 min prior to resolving on a 5% polyacrylamide gel (0.5x TBE) at ~10 V/cm for 2-4 hours at 4^o^C. Supershift assays were performed using antibody cocktails specific to the indicated NF-κB subunits. Competition was performed by pre-incubating the mixture with cold specific and non-specific probe.

### Statistical analysis

Results are expressed as mean ±SEM or SD where indicated. Statistical significance was taken as p<0.05 using a 2-tailed Student's *t*-test. For observable age-related changes, Pearson's chi-square test was used. Kaplan-Meier survival curves were analyzed by the Log-rank method.

## SUPPLEMENTAl FIGURES


