# Supplementary material for: Loss of Nfkb1 leads to early onset aging
Source: Aging (Albany NY). 2014 Dec 1;6(11):931–42. doi: 10.18632/aging.100702 (PMC4276787; doi:10.18632/aging.100702)
Supplement: Supplementary file 1 [file aging-06-931-s001.pdf]

SUPPLEMETAL FIGURES

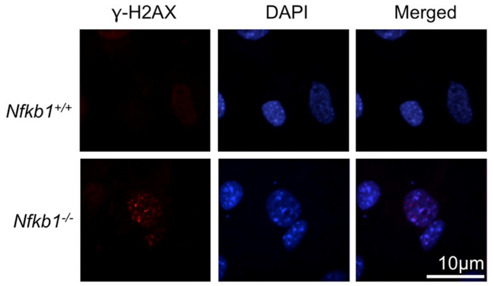

Figure S1. *Nfkb1$^{-/-}$* MEFs accumulate more γH2AX foci than *Nfkb1$^{+/+}$*. Representative immunofluorescence images of γH2AX foci in primary *Nfkb1$^{+/+}$* and *Nfkb1$^{-/-}$* MEFs at passage 4.

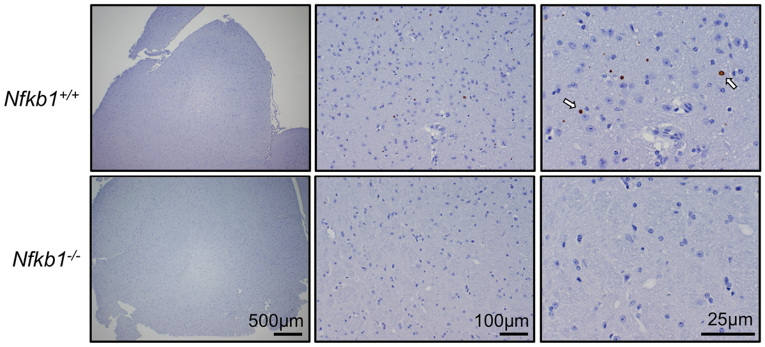

Figure S2. Loss of *Nfkb1$^{-/-}$* results in decreased apoptosis in brain tissue *in vivo*. Representative images of caspase 3 staining of brain tissue from 12-month old *Nfkb1$^{+/+}$* and *Nfkb1$^{-/-}$* mouse brains. Arrows represent caspase 3 positive cells. n=3 per group.

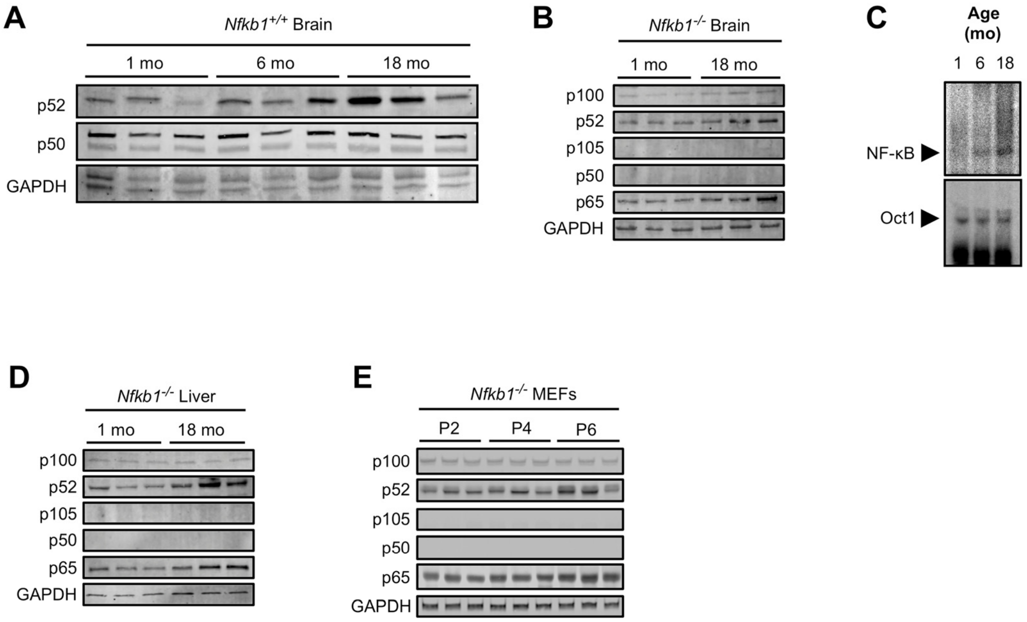

Figure S3. p50 DNA binding declines with age. (**A** and **B**) Immunoblots, using the indicated antibodies, of tissue lysates from (**A**) *Nfkb1$^{+/+}$* and (**B**) *Nfkb1$^{-/-}$* mouse brains at the specified age. (**C**) NF-κB and Oct1 EMSA in brains from 1-month old, 6-month old and 18-month old *Nfkb1$^{+/+}$* mice. Oct1 EMSA demonstrates equal lysate loading. (**D**) Immunoblot using the indicated antibodies in lysates from *Nfkb1$^{-/-}$* mouse liver at the specified age. (**E**) Immunoblot from *Nfkb1$^{-/-}$* MEF lysates at passage 2, 4, and 6. Each lane represents an individual MEF isolate, harvested at different times.
